# Navigating COP16’s digital sequence information outcomes: What researchers need to do in practice

**DOI:** 10.1016/j.patter.2025.101208

**Published:** 2025-03-14

**Authors:** Melania Muñoz-García, DSI Scientific Network, K.C. Bansal, Yiming Bao, Sarah C. Brinkley, Elena Buzan, David Castle, Martha Lucía Cepeda-Hernández, Solenne Correard, Andrew J. Crawford, Jessica M. da Silva, Manuela da Silva, Sonigitu Ekpe, Elmostafa El Fahime, Annika Engelhardt, Davide Faggionato, Aylin S. Haas, Desiree M. Hautea, Martine Hossaert-McKey, Camila J. Mazzoni, Marcel Jaspars, Sally Katee, John Kress, Alexander Kwarteng, Darío A. Lijtmaer, Seon Lee, Isabel Lopez Noriega, Christopher Lyal, Gyanpriya Maharaj, Ann M. McCartney, Douglas Miano, Joseph Mulema, Guilherme Oliveira, Francis Osiemo Omesa, Pablo Orozco, Jörg Overmann, Anna Poetsch, Christine Prat, Débora S. Raposo, Silvia Restrepo, Fabian Rhoden, Mathieu Rouard, Mahloro Hope Serepa-Dlamini, María Alejandra Sierra Aguilera, Mutsuaki Suzuki, Christian Keambou Tiambo, Martin Wiemers, Linda Wong, Eizadora Yu, Maria Mercedes Zambrano, Jinfeng Zhou, Amber Hartman Scholz

**Affiliations:** 1 Leibniz-Institute DSMZ German Collection of Microorganisms and Cell Cultures, Braunschweig, Germany; 2 DSI Scientific Network

## Abstract

The UN Convention on Biological Diversity adopted new rules for sharing benefits from publicly available genetic sequence data, also known as digital sequence information (DSI). In this Opinion, the authors describe the key elements researchers need to be aware of, address real-life questions, and explain the practical implications of these rules for research and development.

## Main text

### Introduction

Since the 13^th^ Conference of the Parties (COP13), in 2016, Parties to the United Nations (UN) Convention on Biological Diversity (CBD) have been trying to solve the “DSI dilemma”: whether and, more recently, how to ensure that benefits from the use of genetic sequence data are shared with countries of origin. The term “digital sequence information on genetic resources” (or “DSI” for short) emerged from early policy debates. It is broadly understood to refer to genetic sequence data and other related molecular biological data, but its exact scope is still undefined. Box 1 defines this and other key terms used in DSI negotiations.

Box 1 **Decoding terms and acronyms**

**Digital sequence information (DSI):** Policy term that refers broadly to genetic sequence data and other related digital data. There is no consensus on a definition, but it is understood to include DNA and RNA sequences and potentially other biological molecular data such as protein sequences, metabolites, and biological structures.

**Genetic resource (GR):** Any material of plant, animal, microbial, or other origin containing functional units of heredity, with actual or potential value. Human GRs are out of the scope of the Convention on Biological Diversity (CBD).

**Associated traditional knowledge (aTK):** Refers to knowledge, innovation, practices, and technologies developed by Indigenous peoples and local communities (IPLCs). There is not a definition under the CBD or the Nagoya Protocol (NP) and the definition varies across national legislation.

**Access and benefit-sharing (ABS):** Principle of the CBD and its NP and other UN instruments that refers to asking permission to a national provider (e.g., government) to access genetic resources and/or aTK and share benefits back to the country where those were collected. This often requires legal contracts (e.g., permits) in which terms of access and benefits to be shared are agreed upon. The benefits could be monetary (e.g., royalties) or non-monetary (e.g., scientific training).

**Non-monetary benefits:** In-kind benefits such as information (research results, reports, scientific papers, others), capacity development or training, and transfer of new technologies to local researchers, communities, and/or authorities. These kinds of benefits have an associated cost but money is not given to the providers of the GR/aTK.

**Conference of the Parties (COP):** Meeting where representatives of all the countries that are Party to an international instrument adopt decisions on its corresponding implementation. The CBD-COP meets every two years and adopts decisions by consensus. The COP decisions are not legally binding but reflect the interests and expectations of the Parties.

**FAIR Principles** for scientific data: FAIR stands for findable, accessible, interoperable, and reusable. These principles were developed to improve the management and sharing of digital data, ensuring that research outputs can be easily found and used.^1^

**CARE Principles** for Indigenous data governance: CARE stands for collective benefit, authority to control, responsibility, and ethics. These principles were developed to encourage that data governance practices respect Indigenous peoples’ rights to control and access their data within big data environments and benefit from its use.^1^

**TRUST Principles** for digital repositories: TRUST stands for transparency, responsibility, user focus, sustainability, and technology. The TRUST principles complement the FAIR and CARE principles by emphasizing the long-term trustworthiness of data repositories, ensuring that data remains accessible, well-managed, and secure over time.^2^

DSI is publicly (and freely) available in open access databases such as those within the International Nucleotide Sequence Database Collaboration (INSDC), and technological and scientific advances, such as synthetic biology, gene editing, and artificial intelligence, enable the development of commercial products without requiring access to physical genetic resources (GRs). Consequently, some policymakers argued these open databases create a loophole to the access and benefit-sharing (ABS) framework under the CBD and its Nagoya Protocol (NP), which requires benefits to be shared back to the countries where the GR originated.

By COP14 in 2018, calls for benefit-sharing from the use of DSI became louder. However, scientists that had experience with the bilateral ABS system under the NP, which is based on national permits to access GR, argued that such a system would be incompatible with the “big data” practices of DSI in modern research. Undeniably, requiring millions of users to obtain individual permits (which often take months or for which legal measures remain unclear^3^) for thousands or millions of sequences would quickly become impractical, if not impossible. Members of the scientific community have actively engaged in CBD international negotiations, seeking compromise between the call for benefit-sharing and compatibility with research and development practices. A multilateral approach, rather than a bilateral one, could maintain open access to DSI while ensuring benefit-sharing, supporting scientific research, and contributing to conservation and sustainable use of biodiversity.^4^

At COP15, following extensive discussions on policy options for DSI, Parties to the CBD decided to establish a multilateral mechanism (MLM) for benefit-sharing arising from the use of DSI and a global fund, and they recognized open access as one of the key principles for the MLM.^5^ In November 2024, during COP16, the CBD adopted the modalities for the MLM and the Cali Fund, defining more details on how benefits from the use of DSI should be shared. In February 2025, at the COP16 resumed session, the Cali Fund was officially launched, enabling businesses that benefit from DSI to start sending contributions. In this Opinion, we explore the practical implications of the CBD-COP decision 16/2^6^ (hereafter “DSI decision”), focusing on academic researchers. We include references to specific paragraphs in the annex to the decision (hereafter just “paragraphs”) to aid readers seeking a deeper understanding of the decision.

### The DSI MLM in a nutshell

The MLM covers all publicly available DSI unless (future) benefit-sharing measures under other ABS instruments come into force (paragraph 1). Other international ABS instruments discussing benefit-sharing rules for DSI are the International Treaty on Plant Genetic Resources for Food and Agriculture (ITPGRFA), under the Food and Agriculture Organization (FAO); the UN Convention on the Law of the Sea on the conservation and sustainable use of marine biological diversity of areas beyond national jurisdiction agreement (BBNJ Agreement); and World Health Organization’s convention, agreement, or other international instrument on pandemic prevention, preparedness, and response (WHO CA+), which includes pathogens ABS (PABS) rules. But those mechanisms are not in place yet and the corresponding UN Fora could choose the CBD-MLM for this end or develop their own mechanism (paragraph 1). A brief description of these international ABS instruments is provided in Box 2.

Box 2 **International ABS instruments**

International legally binding United Nations (UN) conventions, treaties, or agreements that include access and benefit-sharing (ABS) rules for genetic resources (GRs) under their corresponding scopes. Those are:

**Convention on Biological Diversity (CBD):** Objectives are conservation of biodiversity, its sustainable use, and the fair and equitable sharing of the benefits arising out of the utilization of genetic resources.

**Nagoya Protocol (NP):** Agreement under the CBD to implement its third objective on ABS.

**International Treaty on Plant Genetic Resources for Food and Agriculture (IPTGRFA), under the Food and Agriculture Organization (FAO):** Establishes a multilateral system (MLS) with standardized material transfer agreements to ensure benefit-sharing from the use of plant genetic resources included in the annex of the treaty. Whether DSI will be included in the ITPGRFA MLS is still under negotiation.

**UN Convention on the Law of the Sea on the conservation and sustainable use of marine biological diversity of areas beyond national jurisdiction agreement (BBNJ Agreement):** Includes rules for ABS for marine genetic resources collected in waters beyond national jurisdictions and DSI. Adopted in 2023, but it is not yet in force.

**World Health Organization’s convention, agreement, or other international instrument on pandemic prevention, preparedness, and response (WHO CA+):** Includes pathogens ABS (PABS) rules. This instrument is still under negotiation.

**Figure 1:**
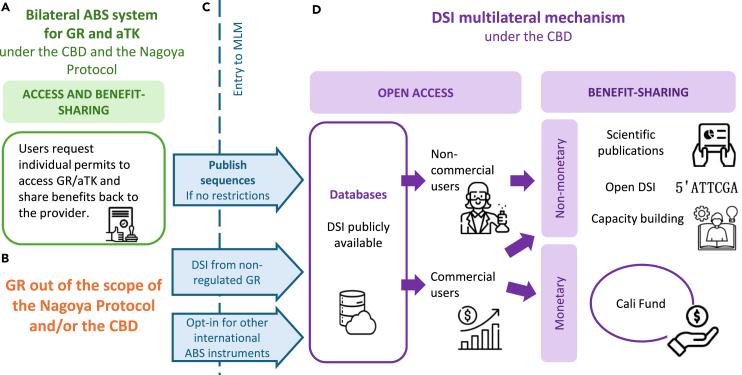
The ABS bilateral system for GR/aTK vs. the CBD DSI MLM Users of genetic resources (GRs) and/or associated traditional knowledge (aTK) must obtain access and benefit-sharing (ABS) permits from the countries where the GR/aTK comes from and share benefits back with that country and/or with the aTK holders (A). The majority of sequences in databases come from non-regulated GR (e.g., from countries that grant free access to their GR) (B). However, once DSI is published in an open access database (C), because it is impractical and inefficient to track and trace who is using which DSI from which country throughout the value chain during research and development, the multilateral mechanism (MLM) decouples access from benefit-sharing (D). Under the MLM, open access remains a key principle, allowing both commercial and non-commercial users to access DSI without needing prior permits, while the MLM modalities, including the Cali Fund, encourages benefit-sharing with Parties. Non-commercial users are expected to share non-monetary benefits and commercial users will be expected to contribute financially to the Cali Fund but will also be encouraged to share non-monetary benefits. Icons source: https://www.flaticon.com/.

In contrast to the NP’s bilateral ABS system, the MLM adopted at COP16 decouples access from benefit-sharing,^7^ preserving open access to DSI and generating benefits that will be shared with countries and Indigenous peoples and local communities (IPLCs), some of the planet’s best custodians of biodiversity.^8^ The DSI decision encourages all users of DSI to share benefits but roles were clarified: non-commercial users should share non-monetary benefits, whereas commercial users are expected to share monetary benefits as well as non-monetary benefits (paragraphs 2, 3, 6, and 9) (Figure 1).

Future contributors to the Cali Fund will be from for-profit entities in sectors that benefit from the use of DSI, such as pharmaceuticals, agribiotech, or cosmetics, and which exceed two of the following three thresholds: total assets of $20 million, sales of $50 million, or profits of $5 million. Such entities should contribute 1% of their profits or 0.1% of their revenue to the Cali Fund. The funds will be directly allocated to Parties using a formula to be determined by COP17. At least 50% of the funds will be allocated to IPLCs (paragraphs 19 and 21).

#### What does it mean in practice?

**Figure 2:**
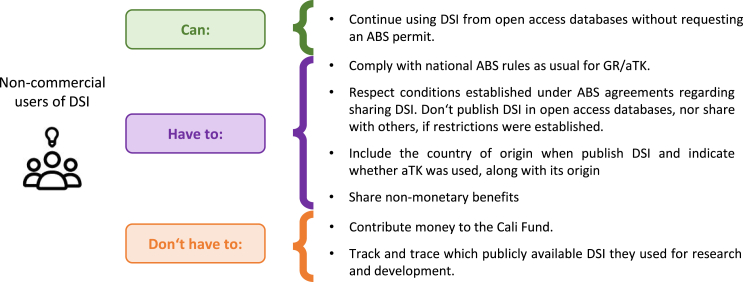
The responsibilities of non-commercial researchers in practice after the DSI decision

In the following sections, we address real-life questions from researchers, clarifying what remains unchanged in the research ecosystem and describing the new modalities scientists need to be aware of (Figure 2).

#### What you can do

Researchers worldwide can continue accessing and using DSI that is publicly available in online databases. COP16 has called on databases to operate in alignment with open access principles, and consider the FAIR Principles for scientific data, the CARE Principles for Indigenous Data Governance**,**^1^ the TRUST Principles for digital repositories,^2^ and the UNESCO Recommendation on Open Science^9^ (paragraph 10.d). There is no need to request an ABS permit to download and use DSI from open access databases. Researchers can also continue submitting and publishing DSI if there are not restrictions on sharing established in the ABS permits obtained for collecting and sequencing the corresponding GRs.

#### What you have to do

Access to GR and associated traditional knowledge (aTK) remains under bilateral ABS national systems. Therefore, you have to obtain an ABS permit or its equivalent to legally utilize these resources, if applicable. Researchers have to check the ABS national measures of the country where the GR/aTK comes from to determine their legal obligations. Research activities have to align with the conditions granted under the ABS permit.

Researchers also must respect the terms and conditions established by the provider country regarding producing and sharing DSI. DSI can continue to be uploaded to open access databases as long as there are no restrictions on making it publicly available. Check your ABS permits for any restrictions to publish DSI in open access databases. To avoid constraints, we strongly recommend considering your DSI generation and publication needs when negotiating ABS agreements, which might also need to align with research funding requirements or journal policies. If a country requests that national clauses be associated to individual DSI in public databases, this would be incompatible with the MLM.

Entities that make DSI publicly available are encouraged to support the implementation of the MLM by notifying users of the new MLM. DSI databases will need to make researchers aware that publishing DSI should be in compliance with applicable ABS obligations and ask for their confirmation that DSI being submitted is not subject to any restrictions that prohibit its sharing (paragraphs 10.b and 10.e).

When submitting DSI, researchers have to include the country of origin of the GR from which DSI was derived. While geolocation of biological samples has been a requirement of the INSDC since 2023, many DSI databases do not yet require this.^10^ COP16 also introduced a new provenance requirement: DSI metadata must also indicate whether aTK was used, along with its origin or source (paragraph 10.c). Discussions are underway to develop traditional knowledge and biocultural labels to be included in DSI metadata.^11^

Finally, as sequences not yet publicly available are not covered by the MLM (paragraph 1), researchers have to check any ABS permit before sharing or receiving sequences not yet published in open access databases, as there may be restrictions and/or benefit-sharing obligations associated with those permits.

#### What you don’t have to do

Non-commercial users of publicly available DSI will not have to contribute monetarily to the Cali Fund, although they may do so voluntarily (paragraph 9). This approach acknowledges the significant role of non-commercial researchers in contributing global knowledge by uploading sequences to open access databases. This practice is not typically followed by commercial users, who often store sequences in private databases, limiting accessibility. Unlike non-commercial users, commercial users will be expected to contribute financially to the Cali Fund and might be obligated to do so in the future under national legislation of the country where they are based.

Users of public DSI don’t have to track and trace which DSI from which countries they use throughout the course of research and development. The DSI benefit-sharing modalities agreed upon at COP16 follow a multilateral approach. Under the MLM, benefit-sharing is based on who uses DSI (academia or commercial sectors) and not on which specific DSI is used. Consequently, contributions to the Cali Fund and non-monetary benefit-sharing will be independent of the country of origin of the GR from which DSI was derived, and users will not be required to demonstrate what DSI they use and its source. The MLM will allocate the funds according to a methodology to be adopted at COP17. This approach avoids legal uncertainties and impracticalities associated with tracking and tracing.

Nevertheless, it may become necessary to keep track of which DSI is under which international ABS instrument, depending on future negotiations under the IPTGRFA, the BBNJ Agreement, and the WHO-PABS and if those decide to establish additional rules for DSI benefit-sharing.^12^

#### Open questions

The DSI decision adopted at COP16 marks a significant milestone, but fully operationalizing the MLM requires further action from various stakeholders. Some of these actions come with a specific mandate for the intersessional period, aiming for adoption at COP17 or COP18, in 2026 and 2028, respectively. Others, however, remain broad and open-ended. In this section, we address the open questions that require further elaboration.

#### Are the MLM modalities adopted during COP16 legally binding?

No, officially, any COP decisions are “soft law” and not legally binding. However, COP decisions express the consensus-based expectations of 196 Parties to the CBD and carry significant political weight. Furthermore, non-binding decisions often serve as a foundation for the development of new policies and, eventually, legally binding agreements. A clear example is the Bonn Guidelines on ABS, adopted in 2002 as a voluntary framework. These guidelines laid the groundwork for negotiating and adopting the NP in 2010.

While negotiating and adopting a new international instrument on benefit-sharing from DSI could take many years, and may never happen, the adoption of the COP16 DSI decision maintains momentum. It advances the policymaking process and enables steps toward receiving benefits from the use of DSI in the near-term.

#### When will the DSI decision enter into force?

The decision became effective immediately upon its adoption on November 1, 2024. However, to fully implement the MLM, several elements must be set up. Parties and other governments need to develop national measures to incentivize commercial users within their jurisdiction to contribute financially to the Cali Fund (paragraph 26). No specific timeline or deadline has been set for these measures, so DSI users should stay informed about any new national regulations.

In the meantime, a steering committee and secretariat for the MLM will be established by the Parties before the next CBD COP. The Cali Fund must also be fully operationalized, and the methodology for allocating funds to Parties should be adopted at COP17. Additionally, Parties are expected to designate a national fund or entity to receive the funds from the Cali Fund and report on their use. The MLM will be reviewed and adjusted at COP18 (paragraphs 19, 20, 28, and 29).

#### What non-monetary benefits do I have to share and how?

In addition to the monetary contributions to the Cali Fund, the DSI decision includes elements for non-monetary benefit-sharing. It highlights the importance of supporting capacity development to produce and use DSI to reduce the gap among countries to benefit from DSI and support national decisions on conservation and sustainable use of biodiversity, food security, and public health and foster their bioeconomies.

An existing clearing house will facilitate non-monetary benefit-sharing, primarily by serving as a match-making platform for capacity-building opportunities. However, details about which clearing house will be used and the specific modalities remain undefined. Additionally, a portion of the Cali Fund may be set aside to support capacity building, but the percentage and the allocation methodology were not agreed during COP16 (paragraph 22).

It is not clear whether and how other kinds of non-monetary benefits, such as open access to DSI, open publications, and scientific collaborations, will be considered under the MLM and how they will be facilitated and measured (paragraphs 7, 8, and 30). Parties could recognize open access to DSI and to scientific data as non-monetary benefits as is done so in article 14.2 of the BBNJ Agreement,^13^ but CBD COP16 left this question undefined.

#### How will national measures on ABS and DSI work alongside the MLM?

This issue is probably the greatest concern among the scientific community and one of the most challenging negotiation points during COP16 and preceding years. In this regard, the DSI decision recognizes national sovereignty by calling on Parties to implement the MLM modalities in a way that is consistent with national legislation and incentivizes participation in the MLM (paragraph 13).

The DSI decision invites Parties to align their national measures with the MLM modalities, aiming to avoid overlapping benefit-sharing obligations (e.g., double payments to both national funds and the Cali Fund) but also retains the phrase “without prejudice to national legislation” (paragraph 26). Therefore, it will be necessary to wait and observe how Parties implement the DSI decision, to what extent the MLM delivers both monetary and non-monetary benefits, and how national legislations evolve in response.

In this context, we hope the MLM will maintain one single set of rules for benefit-sharing from DSI and avoid introducing exceptions at the national level. A proliferation of non-standardized national exceptions to the MLM will increase complexity, raise transaction costs, and incentivize jurisdiction shopping, undermining the efficiency of the MLM.

#### What about DSI covered by other international ABS instruments?

The CBD-MLM covers DSI of all GR, including those GR that would fall under the scope of the BBNJ Agreement and the ITPGRFA, until the moment those instruments put in place their own benefit-sharing modalities for DSI or decide to recognize the CBD-MLM for that purpose (paragraph 1.c).

In this regard, we advocate for harmonized rules for benefit-sharing across existing and future ABS instruments. Aligned or shared modalities among different UN Fora would preserve open access, ensure data interoperability across databases, and enhance benefit-sharing while fostering research and innovation.^12^ The COP16 DSI decision supports this approach, calling for a mutually supportive and adaptive MLM that avoids overlapping benefit-sharing obligations and promotes efficiency. It also invites other UN Fora to collaborate with the CBD-MLM framework to achieve these goals (paragraphs 1.c and 27).

#### The scientific community should remain actively engaged

While COP16 marked a milestone, negotiations on benefit-sharing from DSI are far from over. Upcoming intersessional work and COP negotiations will focus on full operationalization of the MLM and the Cali Fund, as well as the exploration of potential additional modalities for the MLM.^6^ Continued involvement from the scientific community is essential to ensure that decisions made in international fora support, rather than hinder, research, development, and innovation, while respecting countries’ sovereignty and the rights of aTK holders.

## Consortia

DSI Scientific Network consortium authors include (in alphabetical order) K.C. Bansal, Yiming Bao, Sarah C. Brinkley, Elena Buzan, David Castle, Martha Lucía Cepeda-Hernández, Solenne Correard, Andrew J. Crawford, Jessica M. da Silva, Manuela da Silva, Sonigitu Ekpe, Elmostafa El Fahime, Annika Engelhardt, Davide Faggionato, Aylin S. Haas, Desiree M. Hautea, Martine Hossaert-McKey, Camila J. Mazzoni, Marcel Jaspars, Sally Katee, John Kress, Alexander Kwarteng, Darío A. Lijtmaer, Seon Lee, Isabel Lopez Noriega, Christopher Lyal, Gyanpriya Maharaj, Ann M. McCartney, Douglas Miano, Joseph Mulema, Guilherme Oliveira, Francis Osiemo Omesa, Pablo Orozco, Jörg Overmann, Anna Poetsch, Christine Prat, Débora S. Raposo, Silvia Restrepo, Fabian Rhoden, Mathieu Rouard, Mahloro Hope Serepa-Dlamini, María Alejandra Sierra Aguilera, Mutsuaki Suzuki, Christian Keambou Tiambo, Martin Wiemers, Linda Wong, Eizadora Yu, Maria Mercedes Zambrano, and Jinfeng Zhou. They refined and support the ideas presented in the article but were not involved in drafting.

## Acknowledgments

We thank the members and secretariat of the DSI Scientific Network for helpful discussions. This work was supported by the German Alliance of Scientific Organization’s project **“**Access and Benefit Sharing Information Platform.”

## Declaration of interests

The authors declare no competing interests. Andrew Hufton, the editor-in-chief of *Patterns*, is a volunteer member of the DSI Scientific Network.

## Declaration of generative AI and AI-assisted technologies

During the preparation of this work, M.M.-G used ChatGPT in order to review English grammar and fluency. Some of the recommendations were subsequently considered and incorporated to improve the text. After using this tool, M.M.-G and A.H.S reviewed and edited the content as needed and take full responsibility for the content of the publication.
