# Navigating COP16’s digital sequence information outcomes: What researchers need to do in practice

**DOI:** 10.1016/j.patter.2026.101661

**Published:** 2026-09-02

**Authors:** Melania Muñoz-García, DSI Scientific Network, Amber Hartman Scholz

## Main text

(Patterns *6*, 101208; March 14, 2025)

It has come to the authors’ attention that the acknowledgements section of this article omitted an important funding source for one of the members of the DSI Scientific Network consortium. The omitted statement is as follows: David Castle’s research is supported by the Government of Canada through Genome Canada, Genome British Columbia, and Ontario Genomics (OGI-208, OGI-233) and the New Frontiers in Research Fund (NFRFT-2020-00073). The authors apologize for the omission.

